# Blunt Trauma Associated With Bilateral Diaphragmatic Rupture: A Case Report

**DOI:** 10.3389/fsurg.2021.772913

**Published:** 2021-12-01

**Authors:** Marco Pace, Damiano Vallati, Elena Belloni, Marco Cavallini, Mohsen Ibrahim, Erino Angelo Rendina, Giuseppe Nigri

**Affiliations:** ^1^General Surgery Unit, Department of Medical and Surgical Sciences and Translational Medicine, St. Andrea University Hospital, Sapienza University of Rome, Rome, Italy; ^2^Thoracic Surgery Unit, Department of Medical and Surgical Sciences and Translational Medicine, St. Andrea University Hospital, Sapienza University of Rome, Rome, Italy

**Keywords:** emergency surgery, blunt trauma, bilateral diaphragmatic rupture, COVID-19, repair of diaphragm, case report

## Abstract

**Background:** A bilateral diaphragmatic rupture is a rare event that occurs in cases of blunt thoracic-abdominal trauma.

**Case Presentation:** We report the case of a 56-year-old female patient with pelvic fracture and second-stage bilateral rupture of the diaphragm due to a car accident. After a chest and abdominal contrast-enhanced computed tomography (CT) scan, the patient underwent emergency suturing of the left hemidiaphragm. On postoperative day (POD) 4, a CT scan performed due to the sudden onset of dyspnea revealed rupture of the right hemidiaphragm, which was not detected on the preoperative CT scan. On POD 9, the right hemidiaphragm was repaired with mesh during a right thoracotomy. The patient recovered 14 days after surgery. However, the postoperative course was complicated by an asymptomatic COVID-19 infection that significantly delayed her discharge from the hospital.

**Conclusions:** Difficulties in preoperative diagnosis and treatment, together with the lack of data in the literature, make this type of trauma a challenge for all acute care and general surgeons.

## Introduction

Traumatic diaphragmatic rupture (TDR) is a rare event that follows a thoracic-abdominal blunt trauma, particularly in cases of motor vehicle collisions (1). The incidence of TDR is estimated to be between 0.8 and 8% (1), and it is considered a marker of severe trauma (2). TDR occurs more frequently on the left side of the diaphragm (60–70%), resulting in a concomitant herniation of the stomach, spleen, omentum, and colon (3). In contrast, lesions on the right side are less frequent (15–24%) due to the protective effect of the liver (4). Moreover, there is a point of weakness on the left side, the lumbocostal trigone (5). Bilateral rupture which occurs in only 3% of TDR cases, is a relatively rare event with higher mortality rates (1). We report a case of bilateral TDR in which the rupture of the right diaphragm was initially missed.

## Case Presentation

**Figure 1:**
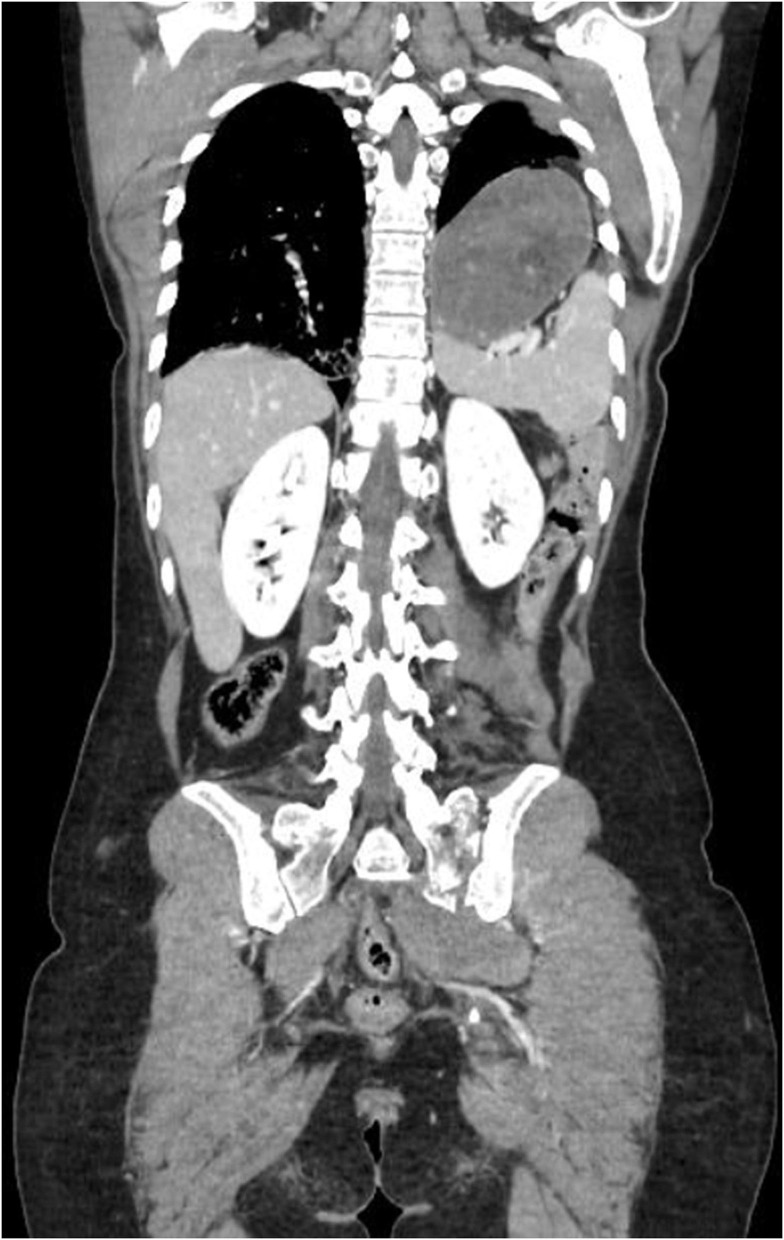
Preoperative Thorax-Abdomen contrast-enhanced CT, showing left postero-lateral diaphragmatic lesion involving the left diaphragmatic pillar and the herniation of the stomach and the spleen in thorax, associated with a lung parenchyma compression and dislocation of the mediastinum.

A 56-year-old woman who was involved in a car accident was admitted to our emergency room with acute abdominal and pelvic high-pressure compressive trauma. She was alert and oriented, tachycardic, and hypertensive. The patient complained of intense dyspnea and thoracic and abdominal pain. Upon inspection, no ecchymoses or hematomas were detected. On thorax auscultation, a vesicular murmur was absent in the left mid-basal hemithorax. The full-body contrast-enhanced computed tomography (CT) scan showed a left posterolateral diaphragmatic lesion involving the left diaphragmatic pillar and a full stomach herniation resulting in lung parenchyma compression and dislocation of the mediastinum to the right (Figure 1). The patient underwent emergent exploratory laparotomy through a bilateral subcostal incision. Abdominal exploration revealed complete herniation of the stomach and spleen into the chest through a 12 cm postero-lateral rupture of the left hemidiaphragm [grade IV, Diaphragm Injury Scale (6)] with the disengagement of the left diaphragmatic pillar. After the stomach and spleen were brought down into the abdomen, the diaphragmatic lesion was sutured with non-absorbable interrupted sutures. No lesions or blood were noted in the suprahepatic region and the Morrison's pouch. A lesion involving the serosa and muscular layers of the transverse colon was repaired using interrupted absorbable sutures. The mucosa was intact and there was no spillage. At the end of the procedure, there were no signs of pneumothorax. Therefore, no pleural drainage was performed.

**Figure 2:**
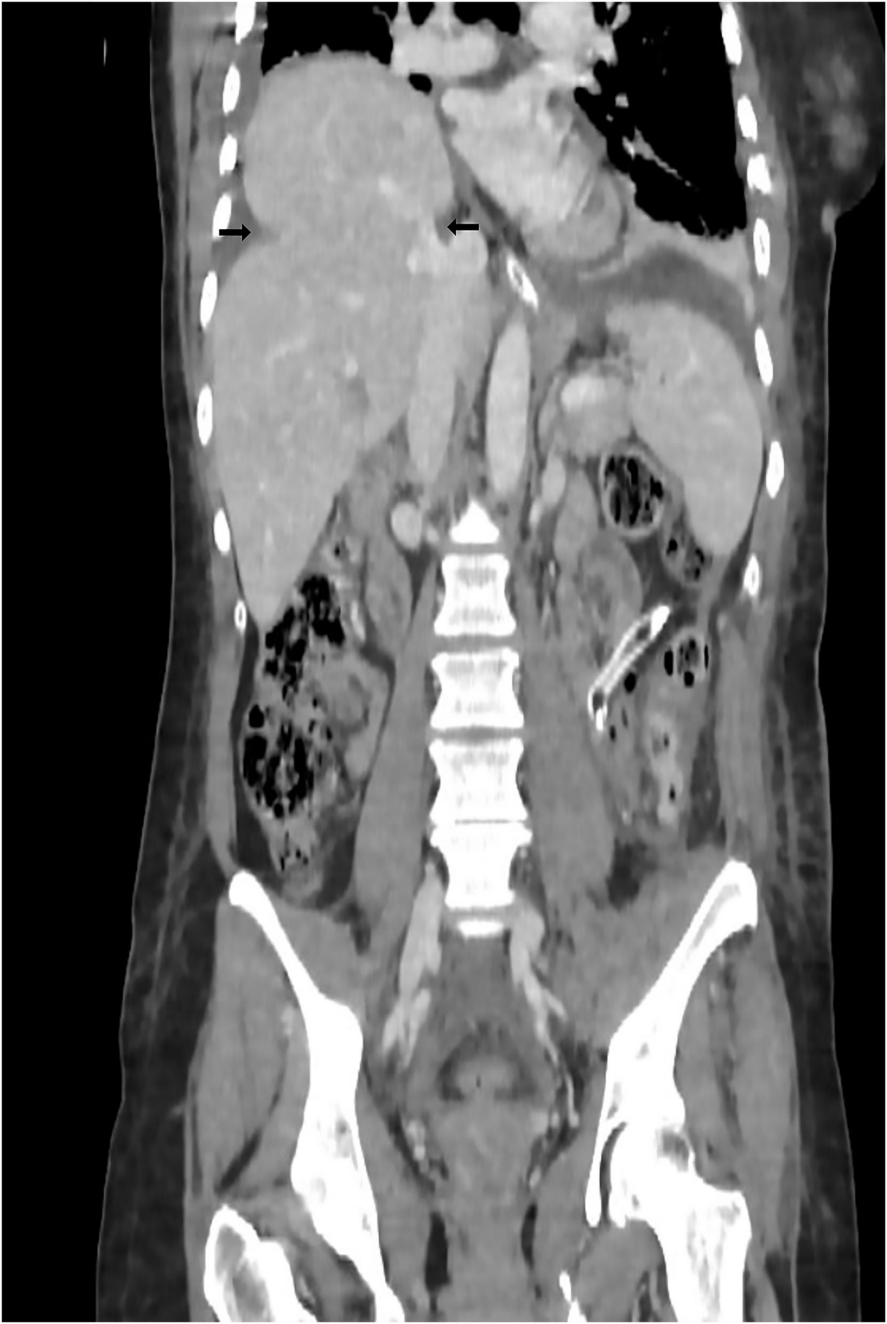
Fourth postoperative day Thorax-Abdomen contrast-enhanced CT that showed the raising of the right liver dome and collar sign (black arrows) with herniation of the VII and VIII liver segments.

On postoperative day (POD) 4, the patient complained of sudden onset of mild dyspnea and chest oppression. A new chest and abdomen contrast-enhanced CT scan showed the right liver dome to be raised and the “collar sign” with herniation of the VII and VIII liver segments through a previously undetected right diaphragmatic breach (Figure 2). The thoracic surgeons were consulted. As soon the patient was deemed stable by the anesthesiologist, the rupture of the right hemidiaphragm was repaired. On POD 9, a posterolateral thoracotomy at the VI intercostal space was performed. With consideration to the delayed onset of the right hemidiaphragm rupture, thoracotomy was preferred to abdominal access (2, 7) as an abdominal access could result in difficulties due to the presence of the liver. A 10 × 7 cm diaphragmatic rupture [grade IV, Diaphragm Injury Scale (6)] was observed with concomitant herniation of the liver segments. The liver was pushed back into the abdomen and the defect was repaired with a DualMesh prosthesis (W. L. Gore & Associates, Inc., Delaware, USA) and interrupted non-absorbable sutures (Video). https://doi.org/10.6084/m9.figshare.16590059. The postoperative course was complicated by non-symptomatic coronavirus disease 2019 infection. This significantly delayed the discharge of the patient.

## Discussion

Bilateral TDR is a very rare event with its incidence reported in only 3% of TDR cases (1). It occurs mainly because of car accidents in which there is a major release of energy. Patients may experience life-threatening injuries to the spleen, kidneys, liver, large and small bowel, lung, thoracic aorta, and abdominal aorta (2.9 vs. 0.2%), as well as multiple fractures of various bones (e.g., pelvic bone, ribs) (1, 4). Although damage to the diaphragm and related complications (herniation and strangulation of the abdominal organs) can be lethal, mortality rates are mainly related to more severe associated injuries (1). Although a delayed diagnosis can increase the mortality and morbidity rates, half of these cases remain undetected, resulting in symptoms such as abdominal pain and respiratory symptoms, years after the acute event (7). Although chest radiography remains the first-level examination, it is diagnostic in only one-third of the cases, compared to chest CT, which is more sensitive (71%) and specific (100%) (4). Chest radiography can recognize diaphragm damage only when the herniated stomach or bowel loops in the chest can be identified (8). Therefore, performing contrast-enhanced CT is mandatory. Through coronal and sagittal CT scans, it is possible to appreciate the pathognomonic signs, namely, the dangling diaphragm sign and the collar sign. The dangling diaphragm sign was first described by Desser et al. as the presence of free edges of the torn diaphragm that take on a comma-shaped appearance and head toward the center of the abdomen (9). The collar sign identifies the imprint of the torn edge of the diaphragm on the herniated organ and is most frequently observed when damage to the left hemidiaphragm causes stomach herniation (8) (Figure 2). Once diagnosed, the surgeon must decide when to intervene and which surgical technique to perform. Currently, there are no precise guidelines on the timing of surgery, and it is usually performed when the symptoms and signs become obvious (10). In particular, when there is herniation of the abdominal organs into the thorax due to the pressure difference between the two cavities, early and aggressive surgical treatment reduces the risk of strangulation and perforation of the herniated organs with consequent increase in morbidity and mortality (2).

Another controversial aspect is the surgical approach. The options available to surgeons are laparotomy, thoracotomy, or both if necessary (2). Laparoscopy and thoracoscopy are rarely used because they require a hemodynamically stable patient and a highly skilled surgeon (4, 7). In instances when TDR is diagnosed early, exploratory laparotomy is recommended. In addition to repairing the damage, exploratory laparotomy allows a wide exploration of the abdominal cavity and exclusion of further injuries of the intra-abdominal organs that may not be detected on CT images (7). For patients with a late diagnosis, a thoracotomy is preferable because it allows for better visualization of the relationships between the herniated organs and the pleural cavity for managing adhesions between the abdominal and thoracic organs (2). In our case, our first approach was to perform a laparotomy to check the integrity of the abdominal organs and repair the defect of the left hemidiaphragm, which allowed the lesion on the colonic wall to be repaired. We avoided exploring the right diaphragmatic dome extensively, because there were no clear images of lesion in the preoperative TC, thus avoiding excessive mobilization of the liver. The damage to the right hemidiaphragm, recognized days after the accident, was treated by thoracotomy. According to other studies, we repaired the damage to the left hemidiaphragm primarily by taking advantage of the pliability of the diaphragm, which is reduced in cases of delayed diagnosis due to fibrotic processes that prevent the rupture from being repaired primarily (2, 4, 11). The possibility of using a prosthesis was not initially considered because of the colonic lesion. Studies on the use of a biological prosthesis in similar cases have been published. The advantages of using a biological prosthesis include a lower risk of infections, adhesion formation, and erosion into surrounding structures (7, 12).

## Conclusion

Bilateral TDR is extremely rare. Given the lack of data, this remains a diagnostic and therapeutic challenge. Although CT provides the most sensitive and specific examination at present, many cases are not diagnosed and remain undetected until they become symptomatic. In terms of treatment options, a gold standard is not available; thus, the choice of treatment is often driven by the experience of the surgeon.

## Data Availability Statement

The datasets presented in this study can be found in online repositories. The names of the repository/repositories and accession number(s) can be found in the article/supplementary material.

## Ethics Statement

Written informed consent was obtained from the individual(s) for the publication of any potentially identifiable images or data included in this article.

## Author Contributions

MP, DV, and EB gathered the data and drafted the manuscript. MI and GN performed the procedures, drafted, and supervised the manuscript. MC and ER supervised the manuscript. All authors approved the final work.

## Conflict of Interest

The authors declare that the research was conducted in the absence of any commercial or financial relationships that could be construed as a potential conflict of interest.

## Publisher's Note

All claims expressed in this article are solely those of the authors and do not necessarily represent those of their affiliated organizations, or those of the publisher, the editors and the reviewers. Any product that may be evaluated in this article, or claim that may be made by its manufacturer, is not guaranteed or endorsed by the publisher.
